# Impact of COVID-19 Pandemic on Daily Life, Physical Exercise, and General Health among Older People with Type 2 Diabetes: A Qualitative Interview Study

**DOI:** 10.3390/ijerph19073986

**Published:** 2022-03-27

**Authors:** Nilton João Chantre Leite, Armando Manuel Mendonça Raimundo, Romeu Duarte Carneiro Mendes, José Francisco Filipe Marmeleira

**Affiliations:** 1 Departamento de Desporto e Saúde, Escola de Saúde e Desenvolvimento Humano, Universidade de Évora, 7000-727 Évora, Portugal; ammr@uevora.pt (A.M.M.R.); jmarmel@uevora.pt (J.F.F.M.); 2 Comprehensive Health Research Centre (CHRC), 7000-727 Évora, Portugal; 3 EPIUnit—Institute of Public Health, University of Porto, 4050-600 Porto, Portugal; romeuduartemendes@gmail.com; 4 Laboratory for Integrative and Translational Research in Population Health (ITR), 4050-600 Porto, Portugal; 5 Northern Region Health Administration, 4000-447 Porto, Portugal

**Keywords:** COVID-19, type 2 diabetes mellitus, physical exercise, health and well-being, qualitative research

## Abstract

The COVID-19 pandemic has resulted in significant alterations to and implications for the lives of millions of people, and especially for those with pre-existing medical conditions. The aim of this study was to explore the lived experience of older people with type 2 diabetes mellitus (T2DM) throughout the first 9 months of the pandemic, with emphasis on the habits of physical exercise. We conducted a qualitative study using semi-structured interviews. The data consist of telephone interviews of seventeen older people with T2DM (10 women and 7 men, aged 62–76 years). Using thematic analysis, five themes were generated: (1) an altered social and relational life; (2) changes in routine and attitude regarding physical activity behaviour; (3) home-related activities gained relevance; (4) health and well-being impact and management; and (5) thoughts about the post-pandemic period. The increase in the number of cases and the fear of becoming infected with COVID-19 limited the social (i.e., contact with family and/or friends) and functional (i.e., daily routine, the habit of exercising) lives of these people, reverberating negatively on their health and well-being. Feelings of isolation, loneliness, anxiety were common. The findings of this study help to better understand the impact of the pandemic and determine areas of need for future interventions. A multidisciplinary approach is necessary to provide support for older people with T2DM and tackle the negative effect of the pandemic, including the reduction in physical activity.

## 1. Introduction

Since the coronavirus (COVID-19) was announced as being a global pandemic on 11 March 2020, it has become a global catastrophe in terms of health, economy and lifestyle [1]. A few days after the announcement, on 19 March 2020, the Portuguese government declared a quarantine period, during which people were prohibited from leaving their homes (except for essential activities) to contain the spread of the virus. Since then, the population has faced different levels of restrictions (i.e., mobility, social activities) that limited participation in normal daily activities. Consequently, these restrictions may have adversely changed health behaviours (e.g., physical activity, diet, sleep patterns), as reported in normal [2,3] and pathological conditions, including people with diabetes [4].

It is recognised that older people and those with a medical condition such as diabetes are at high risk for severe COVID-19-associated disease [1,5]. The last global report reveals that in 2021, 537 million adults (20–79 years) were living with diabetes. The worldwide prevalence of diabetes is estimated to increase to 643 million people by 2030 and 783 million people by 2045 [6]. This high prevalence is associated with the increase in the risk factors such as the ageing of the population, sedentary behaviours, and excess body weight. Type 2 diabetes mellitus (T2DM) comprises 90–95% of all diabetes cases [7]. Such worrying data confirm this disease as being a significant global challenge that may be exacerbated by the consequences of COVID-19 [8,9,10,11].

Based on studies of large populations, the prevalence of diabetes in COVID-19 varies from 5% [12] to 11% [5], and is linked to the increased viral entry into the cells and a compromised immune system [13]. In Portugal, diabetes is the second most common chronic disease to increase the risk of COVID-19 severity [14]. Six of every 10 individuals with diabetes suffer from one or more conditions, including chronic kidney disease, cardiovascular diseases, chronic obstructive pulmonary disease, obesity and smoking [14]. It is already known that people with T2DM are more prone to severe micro-and macrovascular complications and an increased mortality rate compared to non-T2DM controls [15], and recent evidence shows that this population also has an increased risk of dying in hospital with COVID-19 [16]. Thus, people with T2DM need to be cautious and employ protection measures to avoid contracting the virus. Therefore, COVID-19 restrictions have played a significant role in exacerbating both the environmental and personal barriers for the people following them.

It is well known that the adoption of a healthy lifestyle (i.e., physical exercise) helps to prevent complications, improve insulin action, and benefits glycaemic control in this population [17]. Researchers have conducted studies that have raised concerns about the impact of COVID-19 on different aspects of the lifestyle and health of people with T2DM. Perceiving themselves as being a high risk group, they were found to be more worried about contracting the virus and, due to this, show evidence of negative effects of COVID-19 on psychosocial health (i.e., distress, loneliness, feeling isolated) [18,19], glycaemic control (i.e., glycated haemoglobin, fasting plasma glucose) [9], dietary patterns [18], exercise habits and body composition [19]. However, most of these studies focused on an exploration of the socio-behavioural changes in terms of descriptive data through cross-sectional design. To the best of our knowledge, no qualitative research has yet been published to explore a comprehensive understanding of the impact of the pandemic on the lifestyle exclusively of older people with T2DM, for which any disruptive changes may affect the control of the disease and general health [20]. We found one study that explored this topic, but the authors used a mixed sample of adult patients (42 ± 12.35 years) with type 1 and 2 diabetes mellitus, and covered only the lockdown period [21]. It is important to gain a better understanding of the feelings of older people with T2DM and the ways in which they have dealt with concerns related to the management of the disease, physical exercise behaviour, health and well-being, which, in other study designs, may have remained unexplored.

To this end, the present study was conducted with the intention of exploring the lived experience of older people with T2DM throughout the pandemic period, with special emphasis on the habits of physical exercise.

## 2. Methods

### 2.1. Study Design and Participants

We conducted a qualitative study using semi-structured interviews. Because it was not completely safe to perform face-to-face interviews during this pandemic period, we opted for a telephone-based qualitative interview study. The interviewees were older people with T2DM (>60 years old) selected purposively from a dataset of a community scientific research project at the local university, recruited through community health centres, hospitals, daycare centres, flyers and social media. Audio-recorded verbal consent was obtained at the start of the interviews.

The inclusion criteria for participation in the study were: (1) male or female and aged between 60 and 80 years; (2) diagnosed with T2DM; and (3) being independent. Among the 19 participants contacted, 17 volunteered to participate in the study, and none of them had tested positive for COVID-19 prior to the interview. This study is part of a university project approved by the Ethics Committees of the University of Évora and was conducted in accordance with the Helsinki Declaration.

### 2.2. Interview Guide Development and Content

Initially, two researchers were involved in the construction of the semi-structured interview guide, which was subsequently debated in a group of four researchers to enhance reflexivity, agreement and clarity. The interviews followed a common interview guide, based on a conceptual framework established from the extant literature on the impact of the pandemic in different contexts, where the main topics were predetermined but open-ended questions were used to approach deductive-inductive reasoning.

The interview guide was tested with two participants matching the established study inclusion criteria, which resulted in gaining feedback on the clarity of the questions. A second researcher, who acted as a supervisor, listened and took notes on the pilot interviews. After this process, the two researchers carefully discussed the interviews with the purpose of refining the topic guide, adjusting the questions and enhancing interviewer skills. Finally, the interview guide was formalised, tested with another participant and then approved by the research team. Due to its rich content, we decided to include it in the final sample. The structure of the interview guide addressed four main topics: (1) daily life routine (e.g., Before the pandemic and lockdown period, what was your routine on a normal day of the week?); (2) physical exercise habits (e.g., Before the pandemic and lockdown period, had you taken part in some physical exercise? If yes, can you explain why you performed physical exercise? If not, what were the reasons?); (3) management of the disease and physical and psychosocial health (e.g., How did you experience your diabetes throughout this pandemic situation?); and (4) future priorities and expectations (e.g., We would like to know what you expect for the future, when ‘everything goes back to normal’). In the second topic, specific closed questions (i.e., self-report of the type, duration, intensity and frequency of exercise) were used to explore the physical activity behaviour before and throughout the ongoing pandemic. During the flow of the conversation with each participant, additional probing questions were added to ensure the reliability of the information.

### 2.3. Data Collection

Data were collected between 27 December 2020 and 5 January 2021. Before the interview, the participants were contacted to formalise the invitation, to explain the nature of the interview and to ask for an availability schedule for future communication. On the scheduled date and time, each participant was contacted for the formal interview. After elucidating the aims of the study, verbal consent and permission for recording the call (using a cellphone app) were obtained from each participant. A maximum of three interviews was conducted per day, and the average duration was 35.7 min per interview. All interviews were conducted in Portuguese by the primary researcher, a PhD student. During the interview, additional field notes were taken with the purpose of supporting the primary data and to help the researcher describe and analyse the context of the interview. Data collection was stopped when theoretical saturation was achieved, i.e., the point at which no new information was emerging in the interview. Only selected quotes were translated for the purpose of this study.

### 2.4. Analysis

The recorded interviews were transcribed verbatim with personally identifiable information removed, and sent to participants to ensure content accuracy and validity. Subsequently, the participants were contacted to clarify or add any information to their responses. The transcribed interviews were imported into Nvivo13 software and read several times to gain familiarisation with the data. A reflexive thematic analysis was conducted following the steps outlined by Braun and Clarke [22]. Initially, predetermined topics were used for the general categories and then broken down into subcategories based on the information that emerged from the responses of each participant by processing from deductive to inductive coding. First, two researchers (NL and JM) coded three interviews independently. Then, they met to compare and discuss the coded interviews, resolve inconsistencies and develop the preliminary coding frameworks. During the coding process, the first author moved back and forth from the interview transcripts, the related literature and discussions with another researcher (JM), updating the data categorisation to reflect the patterns identified in the data. Regarding the questions on the frequency and time of engagement in exercise, descriptive statistics (mean, minimum and maximum) were used.

## 3. Results

The study included 10 females (58.8%) and seven males (41.2%). The participants’ mean age and time in education were 69.9 years (SD: 5.0; range 62–78 years) and 7.9 years (SD: 3.9; range 4–15 years), respectively. The mean duration of diabetes was 13.7 years (SD: 8.8; range 1–36 years). The mean length of the interview was 35.7 min (SD: 11.8; range 23–59 min). The demographic characteristics of the participants are detailed in Table 1.

Table 2 shows the participants’ exercise patterns before, during and after the COVID-19 restrictions. According to the quantitative information gathered during the interviews, there was a notable change in physical exercise habits from the pre-pandemic to the pandemic period. During the total lockdown (19 March to 2 May 2020), the amount of weekly exercise was drastically reduced (by ~137 min), which corresponded to more than half of the amount of physical activity performed pre-COVID. In the period following the easing of lockdown measures (post-lockdown 3 May to 17 December), there was a slight increase in weekly minutes spent on physical exercise, but this was still well below the pre-pandemic level. In addition, at the time of the interviews, the participants reported themselves to be engaged in 90.1 min of physical activity weekly, which represented reductions of 61.6%, 7.2%, and 32.1% in physical exercise compared to pre-COVID, lockdown and post-lockdown periods, respectively.

From the thematic analysis of the interviews, we identified five major themes describing the participants’ experiences lived throughout the pandemic period: (1) an altered social and relational life; (2) changes in routine and attitude regarding physical activity behaviour; (3) home-related activities gained relevance; (4) health and well-being impact and management; and (5) thoughts about the post-pandemic period. Findings are summarised in Table 3. The following section discusses each of these themes and its sub-themes along with illustrative participant quotes.

### 3.1. An Altered Social and Relational Life

The analysis showed that the interviewees had significant changes in life, social interactions and concerns about restrictions with close family members. Under this theme, we identified three sub-themes related to: (1) changes in social life participation; (2) limited contact with family members; and (3) more time spent at home.

#### 3.1.1. Changes in Social Life Participation

The majority of participants reported a drastic reduction in exposure and refrained from routine involvement in social life.

*I haven’t been out with my friends or anything... I often went out with friends, to drink tea or something... now I haven’t been*.

(Participant no. 10)

*I stopped going to the daycare centre, I stopped going to the city, I hardly leave the house … I never went to the shopping centre again, I never went there with friends or anyone*.

(Participant no. 1)

#### 3.1.2. Limited Contact with Family Members

The participants expressed great difficulty in adapting to the restriction measures, namely, limiting or avoiding contact with other people, including close family members.

*For me it was not easy at all. Because I am a very affectionate person, I love affection, kissing the kids... and they don’t come*.

(Participant no. 14)

*I stopped seeing my children as frequently as I saw them before, we talked more on the phone. It was a completely different way of being*.

(Participant no. 5)

Similarly, participant no. 6 said: ‘I was the one who raised the kids [grandchildren], they always stayed with me... so, she [the mother of the grandchildren] returned to the country because of the pandemic, because the kids were with me, and there was the problem that grandparents should not stay with the kids’.

#### 3.1.3. More Time Spent at Home

Over half of the interviewed reported that staying at home became part of their daily routine to protect themselves throughout this complicated period.

*I never returned to the same routine, never again. I started to get used to being at home, and I never returned to the same routine... I spend more time at home*.

(Participant no. 10)

*I’m staying more time closed at home. I was not a person who went out a lot [pre-COVID-19], but when I went out, I went out freely*.

(Participant no. 13)

### 3.2. Routine Changes and Attitude Regarding Physical Activity Behaviour

One of the major shifts in the lives of individuals who were physically active was the environmental and personal challenges regarding physical activity behaviour during the pandemic. As a result of the public health recommendations and adaptations to lifestyle, (including staying at home), people experienced a mix of barriers and facilitators towards the practice of physical activity. This theme included four sub-themes: (1) adaptations to maintain an active physical life; (2) fear of COVID and lack of motivation; (3) suspension of the use of exercise centres; and (4) physical activity helping both the body and the mind.

#### 3.2.1. Adaptations to Maintain an Active Physical Life

Participants described the alternatives used to maintain their routines of physical exercise while taking precautions. For most, walking in open and less crowded spaces was the preferred way to exercise due to the feeling of security.

*I live in a geographical area where there are few people outside, limiting the contact with people. By going to the Ecopista route, I felt that I could walk freely and that nothing could happen to me*.

(Participant no. 1)

*I always go to areas that have few people, to the [local place]; it is an area where there is practically nobody…I always wear a mask for protection*.

(Participant no. 16)

For others, performing home-based exercises provided an opportunity to stay physically active.

*They [family members] offered me a stationary bike, and I took advantage of it. I have it in the backyard, and I use it*.

(Participant no. 3)

*I used [during the pandemic] to do physical exercise at home because I have that stationary bicycle. I tried to do some physical exercise*.

(Participant no. 6)

#### 3.2.2. Fear of COVID-19 and Lack of Motivation

The pandemic has made it difficult for people to maintain their usual physical activity routines. The participants expressed fear of exercising outside due to the increase in the number of cases of the disease.

*Here in [city of residence] there have been new and greater contamination problems and, therefore, I have reduced the frequency of going out. So, I even reduced the frequency of walking outside a bit*.

(Participant no. 2)

*Now I have been more sedentary because the situation is very serious, especially here in [city of residence]*.

(Participant no. 11)

*I did not do it [walking outside] because I was afraid to go to the street, to avoid walking there … just to avoid*.

(Participant no. 2)

Furthermore, this insecurity resulted in a lack of motivation to exercise.

*I could go walking, but I’m getting lazier, and I stay more at home. Honestly, I began to protect myself a lot and I get more stuck at home, more at home.’ In the same way, the lack of presence of others engaging in a similar activity resulted in frustration in the practice of physical exercise*.

(Participant no. 10)

*I don’t have anybody to motivate me now. I don’t see others doing it [exercising], and it bothers me to do it alone*.

(Participant no. 12)

#### 3.2.3. Suspension of the Use of Exercise Centres

Due to the public restrictions, some fitness exercise facilities closed, which resulted in a lack of motivation.

*I don’t have much motivation to do this [physical exercise] at home … the gym is closed*.

(Participant no. 5)

*It is difficult to stay at home without these activities. We never had gymnastics again*.

(Participant no. 3)

#### 3.2.4. Physical Activity Helping Both the Body and the Mind

For those interviewed who succeeded in maintaining their previous physical activity habits, participation in physical activity was a way to escape some of the negative consequences of the pandemic (e.g., isolation) and to maintain their health.

*I do exercise to get out of house, to have a little freedom... to stop thinking of bad things*.

(Participant no. 9)

*It [physical exercise] gives me a sense of well-being, not only internal but also in physical terms. It makes me feel good, creates more flexibility in terms of movement*.

(Participant no. 15)

### 3.3. Home-Related Activities Gained Relevance

As a result of the public recommendation to stay at home, in most cases outside activities were restricted to shopping for essential goods and keeping medical appointments. Thus, to decrease the contact between people, daily routines were largely replaced by home-related activities throughout the pandemic. Under this theme we identified three sub-themes: (1) compensatory leisure activities at home; (2) indoor activities became more relevant; and (3) shopping continued to be necessary.

#### 3.3.1. Compensatory Leisure Activities

Almost all the participants described that they sought productive alternatives to compensate for the unusual amount of time spent at home. These participants spent part of their free time in leisure activities such as reading books and newspapers, and gardening activities became more pleasant than before.

*At home, I had the opportunity to read... because I like to read, I had the opportunity to finish read some books that I had been reading for a while... because I had other things and did not have time to read*.

(Participant no. 14)

*I have a beautiful garden. Since the confinement started, my garden is much more beautiful than before, much richer, beautiful, beautiful... due to the extra time I have… Truly, I have a beautiful garden*.

(Participant no. 17)

*I knew I should not go out, so I entertained myself with what I had here at home. I have a yard full of flowers, I always had a place to go to do something, I always had something to do… I didn’t think that “I’m tired of being at home”... I do not think like that because of the yard, because it takes a lot of time to care for*.

(Participant no. 8)

#### 3.3.2. Indoor Activities became More Relevant

Some participants expressed that they increased the time spent on housework.

*Now it’s every day [doing housework], I mean, you clean one thing, you clean others, you get a little rest*.

(Participant no. 4)

*My house is medium in size, and I always like to clean and so I didn’t get bored, I didn’t think that “I’m tired of being at home”.*

(Participant no. 8)

Participant 5 also emphasised: ‘Due to the pandemic I spend more time on domestic activities’.

#### 3.3.3. Shopping Continued to Be Necessary

The majority of respondents stated that they went out of their houses exclusively for shopping for food, medicine, and other essentials.

*I go out every 15 days, shopping, going to the pharmacy or to the doctor when it is necessary. On the other side, most participants shared concerns regarding shopping and tried to choose periods of the day with fewer people*.

(Participant no. 10)

*I try to go to the nearby shops in the area of [city of residence], they are small businesses, I chose the hours when I think that there are not so many people*.

(Participant no. 15)

*I go shopping at periods when there are few people. The supermarket opens at 7:30 am, and that is the time I usually go there*.

(Participant no. 16)

### 3.4. Health and Well-Being Impact and Management

This theme included quotes from the majority of the participants. It was composed of four sub-themes: (1) management of diabetes during the pandemic; (2) physical functioning and changes in weight; (3) changes in sleep and dietary patterns; and (4) old and new mental issues.

#### 3.4.1. Management of Diabetes during the Pandemic

Two-thirds of the patients reported changes in their management of the disease after the pandemic onset.

*I am less active, and my diabetes is not so well controlled…when I am exercising, my diabetes values become lower*.

(Participant no. 3)

*I went there [to the doctor] with the clinical analysis, I was at 8.1%. I think that value was the glycated haemoglobin, and the doctor told me that it was an extreme value. He prescribed me another medication and I start to take one pill at night, which was metformin, but it was not enough. Then he prescribed another medicine to take in the morning at breakfast*.

(Participant no. 8)

This critical period has negatively impacted access to healthcare facilities. The participants started to meet their physicians via telemedicine for advice on treatment or to share concerns related to the disease.

*Now it is only by phone that I have contact with the medical doctor*.

(Participant no. 9)

Participant no. 10 added: ‘They did not want the patients in the hospital [laughs], at least for those patients that have routine appointments to show medical exams to their family doctor... I had some [medical consultations] over the phone during the summer’.

#### 3.4.2. Physical Functioning and Changes in Weight

Only three participants did not mention impaired functional fitness. Severe impairments in physical functioning were reported by participants.

*Even now I went to do a medical test there at a local clinic, I went up the stairs to the second floor, and I got there tired, I get very tired on the stairs*.

(Participant no. 3)

Similarly, Participant no. 14 expressed physical difficulties during activities of daily living: ‘In the normal routine of the house, I must sit 2 to 3 times to be able to continue because the tiredness is extreme. It is very tiring’.

Almost all participants mentioned that they experienced changes in body weight during the COVID-19 pandemic.

*I increased [body weight] a little, a little … 10 kg. I have more weight now than before*.

(Participant no. 9)

*I increased, I increased [body weight] because I did not exercise, I did not walk, I spent a lot of time seated… it [the routine] was eating-sitting-sleeping*.

(Participant no. 11)

#### 3.4.3. Changes in Sleep and Dietary Patterns

Most of the patients saw alterations in their sleep and dietary habits during this critical period.

*I spent many hours awake at night, and sometimes I had to get up and sit at the computer until dawn. Sometimes I return to the bed at 4 or 5 in the morning or more. I mean, I spent a part of the night awake. I do not know why*.

(Participant no. 1)

*Normally I have stable sleep throughout the night, but due to the pandemic situation I had moments of not be able to sleep. Participants reported dietary changes, and, as they spent more time at home, they tended to eat more snacks*.

(Participant no. 15)

*When I had my grandchildren [at home, before COVID-19], I used to make lettuce salad, I did everything well … but now, being alone, it’s: “Do you think that I give myself the job to do it?”*

(Participant no. 14)

Participant no. 6 stated: ‘I can snack on something else because I am at home. If I am not at home, that is not so frequent. It is like this: after some fruit, I will eat this, I will eat that, it’s natural to eat more’.

#### 3.4.4. Old and New Mental Issues

Almost all participants reported psychological health issues such as fear, frustration, anxiety and stress.

*This isolation creates a lot of tiredness, some fears, concerns*.

(Participant no. 16)

*I have less patience, I sleep worse and I have a more anxious state of mind, less patience*.

(Participant no. 5)

For those with a pre-existing mental health problem, the current pandemic situation may have increased their vulnerability. Participant no. 3 reported their mental disturbance: ‘When I had [before COVID-19] these activities [exercise maintenance] at the university I was doing well, I was distracted and I did not think about life. Now I think about the life I had during the war, where many of my colleagues died and others were disabled for the rest of their lives’.

### 3.5. Thoughts about the Post-Pandemic Period

In an attempt to project a future beyond the pandemic, the participants addressed a number of concerns, feelings and desires. This theme consisted of four sub-themes: (1) a desired return to normality; (2) an uncertain future; (3) mixed feelings about the vaccine; and (4) a favourable attitude towards an active life.

#### 3.5.1. A Desired Return to Normality

As a result of the COVID-19 pandemic with its many limitations and fears, most of the interviewees expressed the desire to return to their old routine, where they felt free and without social life concerns.

*I am eager to return to normality, to the activities I used to do, and talking with friends*.

(Participant no. 7)

Participant no. 14 also expected to return rapidly to normality: ‘I would like the affection and kisses to come back, that we could have freedom... because the lack of freedom is a terrible thing’.

#### 3.5.2. An Uncertain Future

More than half of participants reported a sense of uncertainty regarding the future. The rate of transmission, mortality, new variants of the virus and social awareness were all factors that made it difficult for the interviewees to think of a future free of the virus. Given this, for many participants, some of the COVID-19 containment measures, such as social distancing and hygiene care, will remain for some time because the population must be prepared and learn to live with the virus as a simple influenza.

*I do not know when we will be back to normality and if we will be back. I’m not very optimistic about the new variants of the virus*.

(Participant no. 10)

*I think that the containment measures will have to continue, such as the use of masks and social distancing, I think that these [measures] will last for some time.*

(Participant no. 17)

*I would like to believe that things are all wonderful, but they will not be... I see that [the situation] is very complicated, and it will be very problematic. I do not say that it will not come back [to normality] one day, but that it is not my understanding right now*.

(Participant no. 8)

#### 3.5.3. Mixed Feelings about the Vaccine

Vaccines are increasingly discussed by leading global organisations and local governments as a means to overcome the COVID-19 pandemic. It may mark the beginning of a return to normality, especially for people’s health. Due to scientific progress, it has been possible to develop a vaccine within a relatively short time. For the participants, such scientific achievement created hope and, at the same time, uncertainties about its effectiveness to bring about the expected end of the pandemic.

*Now, with the vaccine, I believe in science 100%. It also gave me peace of mind, which reflects inwardly*.

(Participant no. 15)

In contrast, Participant no. 5 stated: ‘I do not believe half of the information they give regarding the vaccines, such as the results and the immunity that they will bring’.

#### 3.5.4. Favourable Attitude towards an Active Life

Although there were mixed feelings regarding the future, all participants alluded to the fact that they were now more predisposed than before to be involved in leisure-related physical activities for an active life.

*When everything goes back to normality I will go for a walk because it was something I wanted. I wanted to walk*.

(Participant no. 17)

*I am eager for this [pandemic] to pass so as to be able to walk freely. I will return to my routines, doing my exercises*.

(Participant no. 5)

## 4. Discussion

The COVID-19 pandemic has resulted in significant alterations to and implications for the lives of almost everyone. Such repercussions in everyday life may have been most evident for those belonging to vulnerable groups of the population. The present study reported the lived experience—including the role of physical exercise—of older people with T2DM throughout the pandemic situation. The findings were summarised into five themes: (1) an altered social and relational life; (2) changes in routine and attitude regarding physical activity behaviour; (3) home-related activities gained relevance; (4) health and well-being impact and management; and (5) thoughts about the post-pandemic period.

Research shows that prior to the pandemic, socialisation and physical activity were common and important for health and well-being [23]. Our findings indicated that, due to the pandemic situation, the participants changed their daily routines, spent more time at home, and had limited interaction with family and friends. This scenario reflects the public measures of social distancing imposed by government authorities, which were some of the most important preventive strategies to contain the spread of the virus [24]. At the time of the study, the counties were divided into four groups according to the level of transmission risk, considering the number of cases per 100,000 inhabitants in the last 14 days—moderate, high (between 240 and 480), very high (between 480 and 960), and extremely high (over 960). Given this criterion, our participants were complying with several massive restrictions (belonging to a high-risk county) decreed by the government, including circulation on public roads between 11:00 p.m. and 5:00 a.m., and limitation of operating hours in commercial establishments (five people per 100 square meters and closing until 10 p.m.). The access to the restaurant sector was limited to groups of no more than six people and at 50% of the establishment capacity. Staying in these establishments was allowed until 1:00 a.m. except 26 December and 1 January, where in-person meals were allowed until 3:30 p.m., and posterior service was only allowed on takeout and home deliveries [25]. The increases in the number of cases of COVID-19 may have discouraged the participants from leaving home and maintaining their habits and routines, which may have induced a negative effect on participants’ health and well-being. To our knowledge, most of the published studies reported data when the initial quarantine and other severe social restrictions were in place around the world and when little was known about the pandemic. For instance, a worldwide multicentre study revealed negative social implications in the lives of the general population [24], and a study in the US confirmed such impacts for those with T2DM compared to pre-pandemic levels [26,27]. In a recent qualitative study, participants with diabetes expressed fears and worries about the possibility of obtaining a COVID-19 infection that could affect their health [21]. The pandemic situation also led to behavioural changes among the majority of our participants (i.e., limitations on out-of-home leisure and social activities). It is widely known that social participation enhances psychological well-being and reduces distress, reinforcing the role of social and relational life in human health [28,29]. In contrast, social disconnectedness—limited contact with others and perceived isolation—and a subjective perception of social resources, puts people at high risk of poor physical and mental health [30,31]. Those who experienced some form of social isolation increased their risk of morbidity, cognitive decline, mental disorder (i.e., depression), mortality and a weakened immune system due to stress exposure [30]. Thus, actions are needed to mitigate these consequences for mental and physical health associated with social withdrawal, especially for those who do not have close family or friends and whose only social contact is outside of the home (i.e., community centres, social care) [32]. Evidence of a negative effect of the containment measures (i.e., social distancing, self-isolation) has been reported specifically for those with T2DM regarding their ability to self-manage their disease [33]. A study from the US reported that the pandemic exacerbated difficulties in the management of the disease, linked to an increased feeling of isolation, wherein 24.7% and 12.6% of the participants with T2DM reported more frequent high glucose levels and increased glucose variability, respectively [27]. Good glycaemic control and plasma glucose concentrations, i.e., HbA1c < 7% and 72 and 144 mg/dL, respectively, reduce the risk of infections that may lead to hospitalisation for T2DM outpatients [34,35]. However, keeping these metabolic parameters stable in the pandemic presents a great challenge for these people [36].

Our study revealed that home becomes a central focus for people with T2DM. Many participants reported that their previous daily routines were replaced with productive home-related activities (i.e., housework, reading, gardening). Scientific evidence suggests that increases in time spent in home activities and other hobbies during the COVID-19 lockdown reduce depressive symptoms and anxiety, and increase life satisfaction [37]. Another study concluded that engagement in gardening activities may be a feasible means of promoting mental health through decreased COVID-19-related distress [38]. Importantly, although our study revealed that the participants adapted to their home life with productive alternatives, it seems that this was not enough to mitigate the negative effects of the current pandemic on mental health, as almost all participants alluded to several psychological perturbations (i.e., stress, anxiety, fear). A qualitative study reached similar findings, whereby T2DM sufferers and the general population reported psychological consequences such as stress, frustration, anxiety and fear [39,40]. It should be highlighted that there is evidence that some factors can have a protective effect on mental health during COVID lockdowns, including engagement in physical activity. Thus, a large decrease in mental health symptoms was seen in people who engaged in more than 30 min per day of exercise or gardening and who increased their work by more than two hours per day [37].

International guidelines for physical activity recommend people to undertake at least 150 min/week of moderate-to-vigorous aerobic exercise spread out over a minimum of 5 days, and moderate-to-vigorous resistance training at least 2–3 days/week on non-consecutive days, complemented with flexibility and balance exercises to delay or prevent the long-term health complications related to diabetes [17]. In the present study, we explored the exercise patterns of the participants before, during and after the COVID-19 restrictions, and saw a great reduction in physical exercise habits during the pandemic, to well below the recommended level. At the onset of the pandemic, this was not an unexpected finding, as it was reported across several populations, including those with a medical condition [41], since total home confinement was the main containment measure adopted to avoid the spread of the virus. This trend continued at the time of data collection, which occurred 7 months after the measures were relaxed. Our finding is in line with the results of Rowlands et al. [42], who used an objective measure (accelerometers) to quantify physical activity levels in older people with T2DM before and during the COVID-19 restrictions. Compared to the pre-pandemic period, the authors reported a significant reduction in overall physical activity levels during the lockdown, even following the easing of the restrictions. This result corroborates the negative impact of the COVID restrictions on physical activity [42]. It is difficult to understand exactly the reasons why people maintained their physical activity at low levels over such long period. Considering recently published studies on the diabetic population [43,44], factors such as the rising number of COVID-19 cases, greater susceptibility to infection, and being part of a group at high risk of COVID-19-related mortality could exacerbate the fear of sustaining participation in physical activity outside the home, including gym classes or walking in public places. Concordant results demonstrated that generalised worries are associated with poorer participation in health-promoting behaviours [45]. The negative health risks associated with physical inactivity, which were exacerbated during social isolation and quarantine periods [46], may have a high impact on older people with T2DM. It is known that prolonged periods of inactivity can compromise metabolic health, independent of achieving the recommended guidelines for exercise [47]. As confirmed above, maintaining physical activity in the context of COVID-19 seems to be challenging for older people with T2DM. Acquiring the habit of easy and accessible exercise is crucial in promoting an active and less sedentary life. In this regard, strategies should be considered, such as home exercise, that do not require large spaces or equipment (i.e., chair squats, sit-ups, Pilates, yoga), tv/online exercise classes, or the use of open outdoor spaces to walk, run or to perform other types of exercise [48]. At the same time, the use of smart devices (i.e., phone apps, wearable sensors) can be a useful option to encourage movement [49]. Thus, efforts directed towards encouraging exercise are necessary to mitigate health-related problems during the COVID-19 pandemic [46,50]. 

Some participants in the study presented sleep disturbances, which, in many cases, were linked directly to COVID-19-related news. Concurring with our findings, a previous study reported that being exposed to information overload about COVID-19 can have a negative impact on the psychological health of T2DM participants [51]. Increases in time spent following news about COVID-19 predict declines in mental health and well-being in adults [37], whereas good sleep habits play an important role in the maintenance and restoration of good heath [52]. Previous studies have shown a negative effect of the pandemic in other domains, such as diet [18] and physical functioning [53]. Consistent with our findings, a number of participants declared that by spending more time at home they increased snack consumption and gained weight. As a consequence, some participants declared difficulty in performing basic tasks (i.e., cleaning the house, climbing stairs). Overall, the findings of a meta-analysis showed that COVID-19 lockdowns resulted in a significant increase in the levels of glycated haemoglobin (0.34%; 95% CI: 0.30–0.38), fasting plasma glucose (7.19 mg/dL; 95% CI: 5.28–9.10) and body mass index (1.13 kg/m^2^; 95% CI: 0.99–1.28) in patients with T2DM [9]. Obesity is a significant risk factor for increased morbidity and mortality in people with T2DM, whereas reduction in body weight or the prevention of weight gain confers significant benefits in glycaemic and blood pressure control [54]. As the pandemic advanced, some participants began to have difficulty in managing their disease and needed medical help with their treatment. The participants reported that, to limit the risk of COVID-19 exposure, their regular clinic appointments were replaced by telemedicine, through audio-visual technology, to provide medical services without being referred to in-person care. This seems to be a very helpful alternative in light of the current pandemic situation, and there is available evidence of its successful results in the reduction of body weight and glycated haemoglobin, and time- and cost-efficiency compared to conventional management strategies in diabetes care [55].

Despite the overwhelming desire of the participants to return to the new normality (i.e., family dynamics, social life participation, outside leisure activities), this seems unlikely until vaccination and therapies are available for group immunity. For the participants, the development of COVID-19 vaccines increased expectations but also worries related to their efficacy. In line with our findings, two cross-sectional studies addressed these issues. One showed that, although most of diabetes patients considered COVID-19 infection to be serious, they have a higher rate of vaccine hesitancy [56]. Factors such as lack of awareness, vaccine efficacy, and safety (side effects) contribute to the reluctance to be vaccinated [56]. The other cross-sectional study found that vaccination remains one of the most effective means to control the pandemic [57]. T2DM patients with no previous history of COVID-19 infection were more likely to be fully vaccinated (i.e., receiving two doses) than those with a previous history of the infection—63.9% versus 14.6%, respectively [57]. The pandemic has led society to rethink the past and to adapt and live in a completely different way. The vaccines will probably play a predominant role in restoring normality, but there are still uncertainties regarding the future. Doubts related to new habits, restrictions and social behaviours, and also to the end of the pandemic, were the main points addressed. Although the T2DM participants continued to resist a gradual return to the old routine, even with the relaxation of social restrictions, with the hope of a normal future life they declared themselves receptive to adopting healthy behavioural measures for a more active life.

### Limitations and Strengths

This study has some limitations that must be considered. As is common in qualitative research, the results were obtained from a relatively small sample of older people with T2DM, and restricted to a specific geographic area. Hence, the findings may not allow for inferential generalisation to other settings within the same and/or the general population. The study does not include valid instruments and clinical variables to assess the participants’ general health status (i.e., physical activity, psychological health, glycated haemoglobin). One should also consider that the interview questions covered a considerable period and different pandemic stages and, therefore, may have led to recall bias. Moreover, the interviews were conducted from 17 December 2020 to 5 January 2021, and, due to the constant changing situation related to COVID-19, it is possible that this study did not cover all aspects experienced by older people with T2DM.

However, the study is strengthened by the timeliness of the data collection (~9 months) and, to the best of our knowledge, is the first research to use a qualitative approach to understand the experience lived by older people with T2DM throughout the pandemic situation. Thus, it covered different periods (i.e., lockdown, post-lockdown) in order to understand the impact of the pandemic. Using a qualitative methodology (interview-based), we obtained rich contextual data that would not be possible by quantitative methods only. Thus, specific beliefs, values and concerns of people belonging to a group at high risk of severe complications (including death) associated with COVID-19 were captured in the present study.

## 5. Conclusions

An altered social and relational life, changed routines and attitudes regarding physical activity behaviour, increased relevance of home-related activities, the impact and management of health and well-being, and thoughts about the post-pandemic period were the five main themes identified from the lived experience of 17 older people with T2DM throughout the pandemic period. The findings showed that ~9 months after the start of the pandemic, older people with T2DM were still isolated, afraid of contracting the virus and presenting low levels of physical exercise. Given the health risks associated with an unhealthy lifestyle, mental health problems and difficulties in the management of the disease reported by the participants, there is a growing need for a multidisciplinary approach to tackle the problem. Government policies should be addressed for older people with T2DM who need particular attention, and strategies should be created to manage emergent psychological issues, to promote a healthy lifestyle, and to manage the control of the disease until the desired normality is reached.

## Figures and Tables

**Table 1:** Demographic characteristics of the participants.

Participant	Gender	Age	Education (years)	Diabetes Duration (years)	Marital Status	Length of Interview (min)
P1	Male	72	4	22	Married	58
P2	Male	70	12	12	Married	30
P3	Male	78	6	10	Married	34
P4	Female	73	9	16	Single	45
P5	Female	67	12	1	Married	23
P6	Female	78	12	10	Married	28
P7	Male	63	4	8	Married	27
P8	Female	68	7	8	Married	32
P9	Female	75	4	5	Married	25
P10	Female	72	12	12	Married	25
P11	Male	67	4	10	Married	27
P12	Male	76	4	30	Married	28
P13	Female	62	6	14	Married	35
P14	Female	67	4	20	Single	59
P15	Female	68	15	4	Married	59
P16	Male	70	14	15	Married	32
P17	Female	62	6	36	Married	37

**Table 2:** Exercise patterns of the participants pre- and during COVID-19.

Variables	Pre-Covid	Lockdown (19 March to 2 May 2020)	Post-Lockdown (3 May to 17 December)	The Time of Interviews (17 December 2020 to 5 January 2021)
Frequency (weekly)	3.4 (2–7)	1.6 (0–7)	2.4 (0–7)	1.9 (0–7)
Minutes (daily)	70.6 (30–120)	23.1 (0–120)	30.2 (0–90)	19.5 (0–78)
Minutes (weekly)	234.4 (90–450)	97.1 (0–420)	132.6 (0–420)	90.1 (0–390)
Type of exercise	Outdoor walking, water aerobics, gym, supervised maintenance exercise, dancing.	Outdoor walking, static bicycle, dancing (tele-sessions).	Outdoor walking, static bicycle, dancing.	Outdoor walking, static bicycle, dancing.

Note: Values are reported as mean (min–max).

**Table 3:** Major themes and sub-themes that emerged from the interviews with the T2DM patients.

Themes	Sub-Themes
An altered social and relational life	Change in social life participation
	Limited contact with family members
	More time spent at home
Routine change and attitude regarding physical activity behaviour	Adaptation for maintaining an active physical life
	Fear of COVID-19 and lack of motivation
	Suspension of the use of exercise centres
	Physical activity helping both the body and the mind
Home-related activities gained relevance	Compensatory leisure activities
	Indoor activities became more relevant
	Shopping continued to be necessary
Health and well-being impact and management	Management of diabetes during the pandemic
	Physical functioning and changes in weight
	Change in sleep and dietary patterns
	Old and new mental issues
Thoughts about the post-pandemic period	A desired return to normality
	An uncertain future
	Favourable attitude towards an active life
	Mixed feelings about the vaccine
